# A Balance Test for Chronic Perilymph Fistula

**DOI:** 10.1155/2012/163691

**Published:** 2012-09-18

**Authors:** Jeremy Hornibrook

**Affiliations:** Department of Otolaryngology, Head and Neck Surgery, Christchurch Hospital, 2 Riccarton Avenue, Christchurch 8011, New Zealand

## Abstract

Perilymph fistula is defined as a leak of perilymph at the oval or round window. It excludes other conditions with “fistula” tests due to a dehiscent semicircular canal from cholesteotoma and the superior canal dehiscence syndrome. First recognized as a complication of stapedectomy, it then became apparent that head trauma and barotraumatic trauma from flying or diving could be a cause. Descriptions of “spontanenous” perilymph fistulas with no trauma history followed. It is likely that most perilymph fistula patients have a congential potential weakness of the otic capsule at the round or oval window. The vestibular symptoms have been assumed to be due to endolymphatic hydrops, but there is poor evidence. Their unilateral disequilibrium, nausea, and subtle cognitive problems suggest they are due to otolith disfunction and that these patients have a specific balance abnormality, unlike subjects with unilateral vestibular hypofuction. In this series of twenty patients with a confirmed fistula a logical simplification of Singleton's “eyes-closed turning” test predicted a PLF in twelve with a trauma history. In four no cause was found. In three a prior traumatic event was later recalled, but one patient had concealed it.

## 1. Introduction

 Perilymph fistula (PLF) has been a contentious topic in otolaryngology for fifty years. The modern meaning of the term is a leak of perilymph at the round or oval window. It excludes other conditions with “fistula” tests such as a canal dehiscence from cholesteatoma and the superior canal dehiscence syndrome. PLF was first recognized as a complication in the early days of stapes mobilization and stapedectomy surgery and attributed mainly to the use of gelatine sponge as a seal around the prosthesis [1]. The symptoms were vertigo, tinnitus and hearing fluctuation, imbalance and aural fullness, and the similarity of these to those of endolymphatic hydrops was postulated. By 1982 Shea [2] was able to claim that with the use of a small prosthesis and natural tissue seal the problem had been solved. In 1968 Fee [3] reported on three patients with an oval window PLF following mild head injury or a series of head injuries without concussion. The predominant symptom was dizziness. In 1970 Stroud and Calceterra [4] introduced the term “spontaneous perilymph fistula” based on four adults whose onset of symptoms was apparently unrelated to a traumatic event.

 The recognition that a PLF could occur without stapes surgery or trauma aroused interest in the phenomenon and the publication of some large institutional series. The first was from the University of Iowa [5]. Ears in a hundred and seventy-seven patients were explored for tinnitus, hearing loss, and vestibular symptoms which were disequilibrium and motion intolerance. Other than stapedectomy the commonest cause was trauma (direct, barotrauma, acoustic) and straining, but in 24% there was no identifiable cause.

  In the “Stanford Experience” over eleven years [6] seventy-eight ears were explored for PLF whose commonest symptom was postural unsteadiness but some were said to have vertigo. 51% had no identifiable cause and were called spontaneous. In the Dartmouth-Hitchcock Medical Centre Experience [7] thirty-five fistulas were diagnosed in thirty-five patients. In 79% of the patients the symptoms began soon after an event involving physical or mechanical stress.

 At the House Ear Clinic over a twelve-year period the ears of eighty-six patients were explored  [8]. The main symptoms were “dizziness” and hearing loss but not tinnitus. Where a fistula was found-one-third had a history of ear surgery or trauma. After fistula repair hearing improvement was unlikely, and the House Ear Clinic advised a very cautious approach to the diagnosis of PLF for hearing loss and particularly in children [9].

 At the University of Texas Southwestern Medical Centre Meyerhof [10] explored the ears and patched their windows in a hundred and twenty patients with a variety of symptoms, including tinnitus and sudden hearing loss. The greatest improvement was in those with a trauma history and imbalance and worst for those with only tinnitus or hearing loss. In the Portland Experience on PLF Black and colleagues [11, 12] found seventy-nine fistulas in ninety ears in patients who nearly all had a mild head injury or a series of head injuries without concussion. Their main symptom was “disequilibrium” (90%), subjective aural symptoms being half as common. “Cognitive dysfunction” was also a feature.

The possibility of PLF in children became a topic of interest [13], particularly in congenitally abnormal ears [14]. Supance and Bluestone [15] reported repairing twenty-nine fistulas in forty-four children's ears. The vestibular symptoms resolved in all but the hearing was unchanged in 86%. The unlikelihood of improving hearing with a PLF repair has been emphasised by others [9, 16]. 

 In 1971 Goodhill [17] advanced a theory of labyrinthine ruptures as a possible cause of sudden deafness associated with exertion or trauma. The two proposed mechanisms were implosive and explosive. “Explosive” would require an increase in cerebrospinal fluid (CSF) pressure transmitted from the internal auditory canal or by the cochlear aqueduct which could rupture the basilar membrane Reissner's membrane, the semicircular canal system, the round window membrane or the annular ligament of the stapes. Conversely an “implosive” force from a valsalva manoeuvre causes sudden air pressure through the Eustachian tube, a sharp increase in intratympanic pressure and rupture of the round window membrane or annular ligament of the stapes. Tonkin and Fagan [18] reported on thirteen patients with a round window fistula where the initiating event appeared to be direct head trauma in four, but exertion, barotrauma from flying and diving, acoustic trauma, vomiting and postoperative in the remainder. In The Royal Ear Nose and Throat Hospital in London in a seven-year period thirty-two patients had a confirmed PLF [19]. When the cause was blunt, head trauma of the fistula was always at the oval window. When the cause was barotrauma, exertion or unknown, it was always at the round window. These findings give some credence for Goodhill's theory to explain a round window PLF, but suggest that for an oval window PLF something else is required.

Early temporal bone studies in the 1930s showed that a crack between the round window niche and the posterior canal ampulla was not uncommon but assumed to be artifact. Subsequently it has been shown to be developmental [20]. These findings were the impetus for Kohut's temporal bone studies on patients who *might *have had a PLF [21]. On the *assumption* that PLF ears would have endolymphatic hydrops the paired temporal bones of patients with histological hydrops and from patients with normal hearing and no vestibular symptoms were examined in regard to oval and round window features. In all the normal bones the fissula ante fenestram was closed by cartilage and the round window fissure was sealed by collagen or bone. In the bones with hydrops one had a patent round window fissure and a history of vertigo attacks which had been diagnosed as Meniere's disease. One had a “patent” fissula ante fenestram containing only fibrous tissue and a patent round window fissure and a history of “waxing and waning disequilibrium” that could have been a PLF.

 Early temporal bone studies had shown that a potential patency of the fissula can be present at birth. Kohut suggested that a “patent” fissula ante fenestram could be a preexisting congenital feature predisposing to a PLF. Unfortunately there is an almost total absence of detailed cases of post-mortem histology on ears with a premortem diagnosis of a treated PLF. The only one has been provided by Kohut et al. [22]. A sixty-eight-year-old male with “constant disequilibrium” had hearing loss in the right ear after a five inch gun fired beside it. Sound and sneezing would make him stagger. Exploration of his right ear revealed a PLF at the fissula ante fenestram and round window fissure which were repaired with connective tissue. His balance returned but not the hearing. Four years later his temporal bones were examined. In the unoperated ear the fissula ante fenestram and round window fissure were not potentially patent. In the operated ear both were “patent”. Of particular note there was no evidence of endolympahtic hydrops in either ear.

 The most predominant symptoms of PLF are vestibular, and these have been assumed to be attributable to endolymphatic hydrops in the fistula ear [1]. In animal models of PLF caused by removing or breaching the round window membrane in guinea pigs and cats histology and auditory brainstem thresholds suggest that PLFs can heal, that there may be no long-term hearing loss, and sometimes cochlear hydrops is observed. Electrocochleography (EcochG) with a click stimulus has been used to diagnose hydrops in PLF patients and in guinea pigs, but it is now known that this is a very unreliable criterion. Also it has not been proved that even if hydrops is present that it is the cause of the vestibular symptoms of a PLF [22]. 

 As the vestibular symptoms of PLF are the most predominant, potentially correctable tests of balance and possible provocative stimuli are of interest. An early attempt on the use of ENG testing for eliciting nystagmus by canal pressure with a pneumatic otoscope (Hennebert's sign) predicted a PLF in some patients [23]. This implies stimulation of the vestibuloocular reflex so the stimulus was transmitted to the horizontal canal receptor, presumably requiring a large defect. Black and colleagues used sinusoidal (300–500 mm H_2_O) ear canal pressure in patients with platform posturography to simulate postural reflexes, reflected by postural sway in PLF patients [24]. 

 In 1929 Tullio [25] showed that loud sounds could induce nystagmus in dogs with surgically fenestrated superior canals and head tilting and leg flexion in pigeons and rabbits with intact labyrinths. The possible relevance of the Tullio phenomenon to PLF diagnosis has been considered. Pyykko and colleagues [26] showed that a low frequency sound induced postural sway in seven patients with a suspected and confirmed PLF, but in none of control subjects with a sensorineural hearing loss. McNeill and colleagues [27] also used a 250 Hz tone in standing patients and found a 77% specificity with a confirmed PLF. Clearly it is not specific for a PLF but it appears to be a logical investigation. As the Tullio phenomenon is stimulating a purely vestibulospinal response it is initiated from the otolith organs.

That PLF patients may have a *unique* balance problem was first suggested by Singleton [28]. For the “eyes-closed turning test” the patient is asked to walk forward with eyes closed and turn quickly one step and stop. A positive test is an inability to stay stable. Twenty-three of twenty-six subjects with a fistula had a positive test which appeared negative in patients with other causes of dizziness. A logical simplification was suggested to the author by Zee [29]. As the test is detecting a lateral instability, the patient (eyes closed or blindfolded) is asked to take two steps sideways.

## 2. Methods

The details of twenty patients (all female) with a confirmed PLF treated over twenty years are summarized in Table 1. The ages at the time of repair ranged from 19 years to 78 years. The range of times from onset of symptoms to exploration and repair was 3 months to 10 years (median 1 year). All had a full ear, nose and throat, and neurootological examination and pure tone audiogram. MRI scanning was done in some cases. In two symptoms began after acute otitis media, even though there was a prior traumatic event. The predominant symptoms were difficulty with balance, nausea, motion intolerance (and sometimes new onset motion sickness), and subtle difficulty with memory. All displayed a subtle unilateral balance instability on at least two components of the “sideways stepping” test. In thirteen there was a well-documented preceding traumatic event: head injury, whiplash, direct blow to the ear, and mastoid surgery. In seven the patient could not initially recall a relevant traumatic event or concealed it. Four other patients had negative explorations (Table 2). 

**Figure 1:**
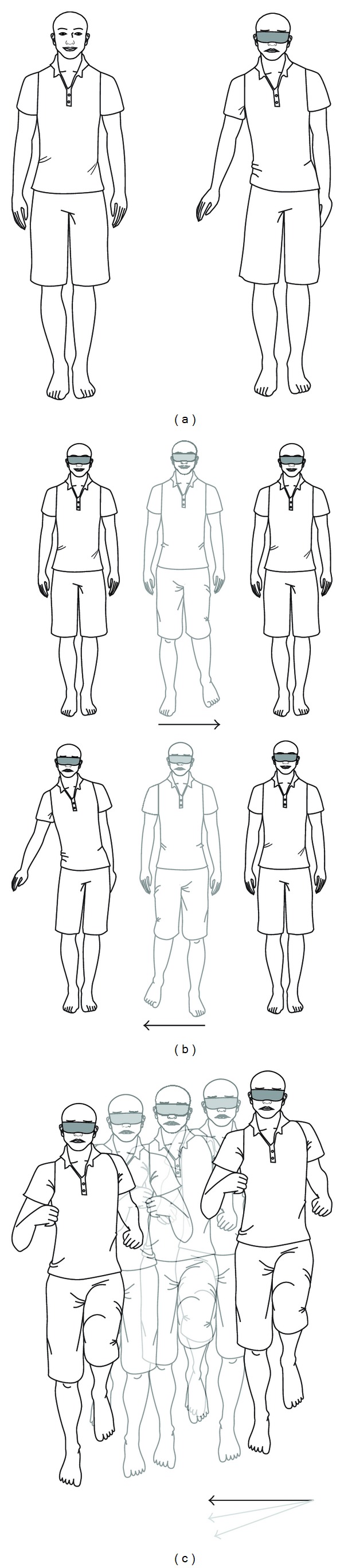
The sideways stepping test. (a) The patient is asked to stand with feet together and hands by the sides. They are then asked to close eyes or are blindfolded. A positive test is an involuntary lean to one side and hip sway. Sometimes the hand is lifted out to compensate. (b) The patient is asked to take two steps sideways and stop, first with eyes open and then with eyes closed or blindfolded. A positive test is an involuntary sway and/or taking a step to compensate. (c) The patient is asked to jog on the spot for 30 seconds with eyes closed or blind-folded. There should be no deviation. A positive test is an involuntary drifting, to one side, and sometimes forward or back. A positive test should be repeatable on subsequent occasions.

 All ears were explored under a general anaesthetic, via an endaural incision. Two drops of optical fluorescein were added to the local anaesthetic [30]. A posterior tympanomeatal flap was elevated. Bone posterior to the chorda tympani nerve was curetted to give maximum exposure of the oval window. The round and oval windows were inspected, and when required the anaesthetist was asked to increase intrathoracic pressure. Mucosa adjacent to the fistula site was elevated and the fistula site packed with connective tissue from the endaural incision and (in most cases) covered with tissue glue. At six weeks the operated ear was inspected, a repeat pure tone audiogram performed, and the patient's balance retested Figure 1.

## 3. Results

Of the twenty-one PLFs seven were in the right ear and fourteen in the left ear (Table 1). Patient 8 with a round window fistula from a direct blow to the left ear had a recurrence eight years (with symptoms for a year) later from a mild head injury. Six PLFs were at the round window. Twelve were at the fissula ante fenestram of the oval window, one with an extra crack in the footplate. Two were at a crack in the footplate. In one round window fistula (patient 10) an air bubble was seen on the other side.

 Postoperatively none had hearing loss attributable to the operation. One required repair of a small eardrum perforation. All had complete recovery of balance, resolution of motion intolerance and nausea, and their subtle cognitive difficulties.

Table 2 lists four patients with negative explorations. Patient 7 rerepresented two years later requesting reexploration, which was negative but remains free of disequilibrium after seventeen years. Twowith no PLF had no change in symptoms. It became apparent that patient 22 had functional imbalance and early dementia. Just prior to exploration she displayed a dramatic instability in all directions.

 Seven confirmed PLF patients were requestioned about a possible traumatic cause (Table 3). In four none could be found. In three an event was found. Patient 1 remembered helping with building repairs and being struck on the head by a ladder and then hitting her head on a plank. Patient 6 was an air hostess. Her hospital records revealed that she had been admitted four years prior with a neck injury sustained when she hit her head on the galley roof as the aircraft plunged in an air pocket. Her symptoms began after acute otitis media. Patient 9 admitted that her husband had “smacked” her left ear and that she had always known that this was the cause. 

## 4. Discussion

In this series the main presenting symptom was vestibular—a persisting subtle abnormality of their balance. Most could nominate a particular side. A particular feature was that they knew, unlike most vestibular disorders, that it was there immediately upon wakening and before moving. It fluctuated, with “good” and “bad” days. On a “bad” day many had mild nausea and an unpleasant aggravation by vehicle motion, without a prior history of motion sickness. Most had subtle cognitive problems, such as inability to remember simple familiar facts and a frustration of “not coping.”

Trauma from head injury, flying and diving barotrauma, sneezing, coughing, and labor as the most common cause of a PLF has been a feature in all the institutional series discussed. Three novel causes have been lightening strike [31], airbag trauma [32], and acoustic trauma from a fire engine siren [33]. 

Grimm and colleagues [34] performed detailed neurological studies on one hundred and two adults with mild defined craniocervical trauma who had a confirmed PLF. The predominant symptom was “disequilibrium, dizziness”, motion intolerance, nausea, memory loss, stiff neck, and headache. Hearing loss was a less common feature. They emphasised that these symptoms could be or easily assumed to be postconcussional syndrome. Grimm [35] has suggested that these subtle symptoms of a PLF make it a neurological syndrome as well as otological. After an inner ear injury there is nearly always recovery or central adaption. However, a PLF is a rare example of an *unstable* peripheral organ [36]. The vestibular system is a very primitive aspect of brain function which preoccupied with calculating gravity and orientation to earth-vertical, so when it is perpetually confused, higher brain function may become subtly involved. There is increasing evidence from other human and animal studies that patients with acute vestibular disorders can also experience cognitive dysfunction [37].

There are numerous descriptions of clinical balance testing on PLF patients, as variations on the Romberg test. The Fukuda/Unterberger test is well accepted as a clinical balance test of vestibular *hypofunction.* References to the fact that PLF patients have a unique imbalance from otolith dysfunction (rather than from hydrops) are rare [38], but it is a valid proposal. 

The terms often used for PLF vestibular symptoms have been “dizziness,” “imbalance,” “disequilibrium,” and often “vertigo.” In contrast to “dizziness” vertigo has always had at its simplest level a well-understood definition of an hallucination of motion, but in the PLF literature the term has been been used loosely and probably to mean any vestibular symptom. If the PLF patient is truly experiencing vertigo, it implies a discrete attack of rotational vertigo caused by Meniere's disease or something resembling it and should be *personally witnessed* by the clinician. With the best of intentions one cannot diagnose a cause of a patient's vestibular symptoms purely from their description of them. Similarly the claim that PLF patients have positionally induced nystagmus may be explained by coincidental benign positional vertigo.

 The Barany Society has sought to refine the definition of common vestibular symptoms [39]. Vertigo is “the sensation of self-motion when no self-motion is occurring or the sensation of distorted self-motion during an otherwise normal head movement.” Dizziness is “the sensation of disturbed spatial orientation without false or distorted sense of motion.”

PLF patients do not describe either of these. The most predominant symptom is of being “off balance” or disequilibrium (the new Barany Society term is “lateral pulsion”). This again raises question that PLF patients have a *unique *balance abnormality that is not explained by hydrops or by vestibular* hypofunction * in the affected ear. There are no VEMP studies on PLF patients, but an abnormal VEMP in a PLF ear may provide (other than possible hydrops) some evidence. 

There is as yet no vestibular condition in which a balance test is the only or the essential diagnostic feature. The accuracy of any balance test can be compromised by patient understanding, concentration, and motive (both intentional and unintentional). Clearly Singleton's “eyes-closed turning test” and the simplified modification described here require objective verification on PLF subjects, normal subjects and in patients with conventional unilateral vestibular hypofunction [40]. 

In summary a PLF is likely to occur in subjects with a potential congenital patency at the round or oval window. The main symptoms are vestibular with a subtle abnormality of balance, suggesting otolith disfunction. Nearly always there is an identifiable prior traumatic event. As other authors have noted it may have been forgotten by the patient and, in fact, even concealed. When there seems not to be, idiopathic PLF is a more appropriate term than spontaneous.

## Figures and Tables

**Table 1:** Twenty patients with a surgically confirmed PLF.

Patient	Sex	Age	Symptoms For	Ear	Site	Repair	Preceding event	Symptoms	Followup
1	F	51	4 yr	Left	OW (FA)	CT	Nil	Disequilibrium to left; nausea; subtle memory difficulty; normal hearing	23 yr

2	F	51	5 mo	Right	RW	CT	Fainted→concussion	Disequilibrium to right; motion intolerance, tinnitus right ear; normal hearing	22 yr

3	F	35	6 mo	Left	OW (FA)	CT	2 whiplash injuries	Acute otitis media→vertigo + vomiting→disequilibrium to left; nausea; motion intolerance; subtle memory difficulty; normal hearing	21 yr

4	F	59	8 mo	Right	OW (FA)	CT	Nil	Disequilibrium to right; nausea; motion intolerance; subtle memory difficulty; normal hearing	20 yr

5	F	40	1 yr	Left	OW (FA)	CT	Face hit by cricket ball; knocked down by a sheep	Vertigo after acute otitis media; disequilibrium to left; nausea; motion intolerance; subtle memory difficulty; normal hearing	20 yr

6	F	37	3 mo	Left	OW (central footplate)	CT	Nil	Acute otitis media→disequilibrium to left; motion sickness; subtle memory difficulty; normal hearing	20 yr

7	F	63	15 yr	Left	OW (FA)	CT	MVA→whiplash	Disequilibrium to left, nausea, motion intolerance, tinnitus and sensorineural hearing loss left ear. Positive Hennerbert's test.	19 yr; see Table 2

8	F	19	6 mo	Left	RW	CT + glue	Struck over left ear by milking cups	Disequilibrium to left; nausea; motion sickness; subtle memory difficulty; normal hearing	17 yr
	F	28	1 yr	Right	RW	CT + glue	Concussion and whiplash	Same symptoms	9 yr

9	F	53	2 yr	Right	RW	CT + glue	Nil	Disequilibrium to right; nausea; motion intolerance; tinnitus right ear; normal hearing	16 yr

10	F	45	1 yr	Left	RW (air bubble)	CT + glue	Nil	Disequilibrium to left; nausea; subtle memory difficulty; normal hearing	16 yr

11	F	34	18 mo	Right	RW	CT + glue	Nil	Disequilibrium to right; nausea; motion sickness; popping tinnitus right ear; normal hearing	15 yr

12	F	40	6 mo	Left	OW (FA)	CT + glue	Punched on left ear	Disequilibrium to left; nausea; motion sickness; tinnitus left ear; normal hearing	13 yr

13	F	40	6 mo	Right	OW (FA)	CT + glue	Nurse. Hit head on bed frame	Disequilibrium to right; nausea; motion sickness; subtle memory difficulty; normal hearing	12 yr

14	F	26	7 yr; worse 9 mo	Left	OW (FA)	CT + glue	Recent head injury; previous whiplash and prior fall from horse→ head injury	Disequilibrium to left; nausea; motion intolerance; tinnitus left ear; normal hearing	11 yr

15	F	42	10 yr	Left	OW (FA)	CT + glue	Whiplash in train crash	Disequilibrium to left; falls; nausea; motion intolerance; normal hearing	9 yr

16	F	78	3 yr	Right	OW (FA)	CT + glue	Fall from a horse; mastoidectomy at age 2 yr	Disequilibrium to right; motion intolerance; mixed hearing loss right ear	7 yr

17	F	63	3 yr	Left	OW (FA) (+crack in footplate)	CT + glue	Nil	Disequilibrium to left; nausea; motion sickness; normal hearing	6 yr

18	F	61	6 mo	Left	RW	CT + glue	Mastoidectomy presenting as meningitis 13 yr prior	Disequilibrium to left; nausea; motion intolerance; tinnitus; left mastoid cavity; no hearing left ear	6 yr

19	F	47	17 mo	Left	OW (FA)	CT + glue	MVA whiplash injury	Disequilibrium to left; nausea; motion intolerance; Nausea from loud sounds; normal hearing	6 yr

20	F	32	8 mo	Left	OW (crack in footplate)	CT + glue	Head injury in fall from horse	Disequilibrium to left; nausea; nausea from vestibular therapy; normal hearing	4 yr later repair small drum perforation.

RW: round window, OW: oval window, FA: fissula ante fenestram, CT: connective tissue.

**Table 2:** Four patients with a negative exploration for PLF.

Patient	Sex	Age	Ear	Symptoms for	Site	Preceding event	Symptoms	Followup
7	F	65 yr	Left	3 mo	No leak	Nil	Disequilibrium to left, motion intolerance	17 yr. dysgeusia. Sensorineural loss increased. Later left BPPV. No disequilibrium

21	F	62 yr	Right	1 yr	No leak	Blew nose	Disequilibrium to right, nausea, sensorineural loss	15 yr. Unchanged

22	F	68 yr	Left	5 yr	No leak	MVA→whiplash	Disequilibrium to left, nausea, poor memory	1 yr. Dementia. Functional imbalance

23	F	28 yr	Left	1 yr	No leak	Nil	Disequilibrium to left, motion intolerance	1 yr. Unchanged

**Table 3:** Eventual cause found in seven confirmed PLF patients without a trauma history.

Patient	Sex	Age	Symptoms for	Ear and Site	Eventual confirmation of traumatic event
1	F	51	4 yr	Left OW	Struck on head by swinging ladder; hit head on a plank
4	F	59	8 mo	Right OW	Not found
6	F	37	3 mo	Left OW	4 yr prior hit head on galley roof in plunging aircraft
9	F	53	2 yr	Right RW	Not found
10	F	45	1 yr	Left RW	Admitted her husband had “smacked” her ear
11	F	34	18 mo	Right RW	Not found
17	F	63	3 yr	Left OW	Not found
